# The Transcription Profile Unveils the Cardio-Protective Effect of Aspalathin against Lipid Toxicity in an In Vitro H9c2 Model

**DOI:** 10.3390/molecules22020219

**Published:** 2017-01-31

**Authors:** Rabia Johnson, Phiwayinkosi V. Dludla, Christo J. F. Muller, Barbara Huisamen, M. Faadiel Essop, Johan Louw

**Affiliations:** 1 Biomedical Research and Innovation Platform (BRIP), Medical Research Council (MRC), Tygerberg 7505, South Africa; pdludla@mrc.ac.za (P.V.D.); christo.muller@mrc.ac.za (C.J.F.M.); bh3@sun.ac.za (B.H.); johan.louw@mrc.ac.za (J.L.); 2 Division of Medical Physiology, Faculty of Health Sciences, Stellenbosch University, Tygerberg 7505, South Africa; 3 Department of Biochemistry and Microbiology, University of Zululand, Kwadlangezwa 3886, South Africa; 4 Cardio-Metabolic Research Group (CMRG), Department of Physiological Sciences, Stellenbosch University, Stellenbosch 7599, South Africa; mfessop@sun.ac.za

**Keywords:** diabetes mellitus, hyperglycemia, cardiomyopathy, lipotoxicity, polyphenols, aspalathin

## Abstract

Aspalathin, a *C*-glucosyl dihydrochalcone, has previously been shown to protect cardiomyocytes against hyperglycemia-induced shifts in substrate preference and subsequent apoptosis. However, the precise gene regulatory network remains to be elucidated. To unravel the mechanism and provide insight into this supposition, the direct effect of aspalathin in an isolated cell-based system, without the influence of any variables, was tested using an H9c2 cardiomyocyte model. Cardiomyocytes were exposed to high glucose (33 mM) for 48 h before post-treatment with or without aspalathin. Thereafter, RNA was extracted and RT^2^ PCR Profiler Arrays were used to profile the expression of 336 genes. Results showed that, 57 genes were differentially regulated in the high glucose or high glucose and aspalathin treated groups. Search Tool for the Retrieval of Interacting Genes/Proteins (STRING) analysis revealed lipid metabolism and molecular transport as the biological processes altered after high glucose treatment, followed by inflammation and apoptosis. Aspalathin was able to modulate key regulators associated with lipid metabolism (*Adipoq*, *Apob*, *CD36*, *Cpt1*, *Pparγ*, *Srebf1/2*, *Scd1* and *Vldlr*), insulin resistance (*Igf1*, *Akt1*, *Pde3* and *Map2k1*), inflammation (*Il3*, *Il6*, *Jak2*, *Lepr*, *Socs3*, and *Tnf13*) and apoptosis (*Bcl2* and *Chuk*). Collectively, our results suggest that aspalathin could reverse metabolic abnormalities by activating *Adipoq* while modulating the expression of *Pparγ* and *Srebf1/2*, decreasing inflammation via *Il6/Jak2* pathway, which together with an observed increased expression of *Bcl2* prevents myocardium apoptosis.

## 1. Introduction

During the last decade, there has been considerable interest in the use of plant-derived polyphenols as nutraceuticals to slow down the progression of metabolic diseases [1,2]. *Aspalathus linearis* (commonly known as rooibos) is a rich source of plant polyphenols with known health promoting properties. In addition to reversing ischemia/reperfusion injury in the isolated perfused rat heart [3], rooibos has been shown to improve both lipotoxicity and oxidative stress in diabetic individuals at risk of developing cardiovascular disease [4,5]. Furthermore, literature has indicated that polyphenols specific to rooibos may present strong ameliorative properties against diabetes mellitus and its associated complications [6,7,8]. Of note, aspalathin, a *C*-glucosyl dihydrochalcone found uniquely in rooibos, has displayed an even greater potency to prevent diabetes-induced cardiovascular complications [9,10,11]. For example, a fermented rooibos extract containing abundant levels of aspalathin protected primary isolated rat cardiomyocytes from experimentally induced oxidative stress and apoptosis [9]. Moreover, recent data from our laboratory demonstrated that aspalathin was able to protect H9c2 cardiomyocytes against high glucose-induced shifts in substrate preference by decreasing fatty acid uptake and oxidation, inferring that aspalathin might act as a fatty acid oxidation modulator in the heart of the diabetic individuals. Furthermore, we demonstrated that aspalathin treatment was able to prevent mitochondrial membrane depolarization and subsequent apoptosis by reducing DNA nick formation, while increasing Bcl2/Bax ratio [10].

Altered substrate metabolism and reduced myocardial production of adenosine triphosphate (ATP) are main contributors to the development of left ventricular dysfunction, a characteristic of the diabetic cardiomyopathy (DCM). Apart from altered substrate metabolism, DCM has been associated with an enhanced intracellular lipid accumulation as observed in hearts obtained from leptin-receptor-deficient diabetic mice (*Lepr*^db/db^) and diabetic patients with cardiomyopathy [12,13,14]. The precise molecular mechanisms associated with the etiology of DCM remains to be fully elucidated. However, gene regulatory pathways of peroxisome proliferator-activated receptor α or γ (*Ppparα/γ*) and sterol regulatory element-binding protein 1c (*Srerbp1c*) have been implicated as important molecular switches that regulate shifts in substrate preference and subsequent lipid accumulation in the heart of diabetic patients [15,16]. Notably, in a study done by Marfella et al. [15], increased expression of *PPARγ* and *Srebf1c* was observed in pressure overload-hearts from diabetic patients. Similarly, in a study done by Son et al. [16], transgenic mice overexpressing *PPARγ* in the myocardium showed increased expression of fatty acid oxidation and lipid storage genes, inferring that hyperglycemia and impaired lipid accumulation play an important role in the development of DCM. It would therefore be of interest to use RT^2^ PCR Profiler Arrays to predict the possible transcriptional mechanisms used by aspalathin to protect the myocardium against the development of high glucose-induced cardiomyopathy. Therefore, an isolated H9c2 model was employed to unravel this mechanism. Results obtained showed that aspalathin protected *Lepr*^db/db^ mice against raised levels of total cholesterol, triglycerides and low-density lipoprotein (LDL) possibly by modulating genes associated with high glucose-induced lipotoxicity, fatty acid oxidation, insulin resistance, inflammation and apoptosis as demonstrated in H9c2 cardiomyocytes.

## 2. Results and Discussion

### 2.1. Effect of Aspalathin on Lipid Profiles

Increased morbidity observed in type 2 diabetes individuals is primarily associated with heart failure, with lipotoxicity being the key pathological mechanism underlying the diabetic condition [5,10,12,14]. The ability of aspalathin to improve lipoprotein clearance was assessed by performing a total lipid profile on mice treated with two dose levels of aspalathin for six weeks (Table 1). The control received no aspalathin. The diabetic untreated control *Lepr*^db/db_UC^ mice had increased body weight, plasma glucose levels (fasting plasma glucose (FPG)), insulin, homeostatic model assessment-insulin resistance (HOMA-IR) levels with associated elevated triglycerides, total cholesterol, LDL and high density lipoprotein (HDL) levels when compared to the nondiabetic lean untreated control *Lepr*^db/+_UC^ group (Table 1). Aspalathin treatment, was able to dose–dependently modulate this condition as the effect of the high dose was greater than either the low dose aspalathin or the metformin treated groups. However, similar to metformin, aspalathin treatment did not affect HDL cholesterol or reduce raised FPG levels. Inferring that aspalathin treatment improves serum lipid profiles and cardiac risk associated with an increased LDL (Table 1). However, it is known that *Lepr*^db/db^ mice display a defective catabolism for the major apolipoprotein A-1 (*Apoa1*), leading to increased HDL and atherosclerosis protection [13]. Similarly, the failure of aspalathin to lower FPG levels can be due to factors such as duration of treatment since previous studies have demonstrated its effectiveness when used at a shorter time interval [7,8]. However, based on our model to induce cardiomyopathy between 9 and 10 weeks, the disease was at an advance stage with chronically elevated blood glucose levels, which resulted in increased oxidative stress and subsequent cardiac damage. Thus, we did not expect either metformin or aspalathin to have an effect on hyperglycemia.

Cardiac failure has been associated with an enhanced intracellular lipid accumulation [12,13,14]. In the current study, we showed that aspalathin treatment significantly lowered plasma lipid levels in *Lepr*^db/db^ mice. To provide insight into the molecular mechanisms by which aspalathin improved lipid profiles in *Lepr*^db/db^ mice, an isolated in vitro H9c2 cell-based system was used to investigate the effect of aspalathin on lipotoxicity in the heart. All subsequent gene expression profiling experiments were done using this model.

### 2.2. In Vitro Effects of Aspalathin

**Figure 1:**
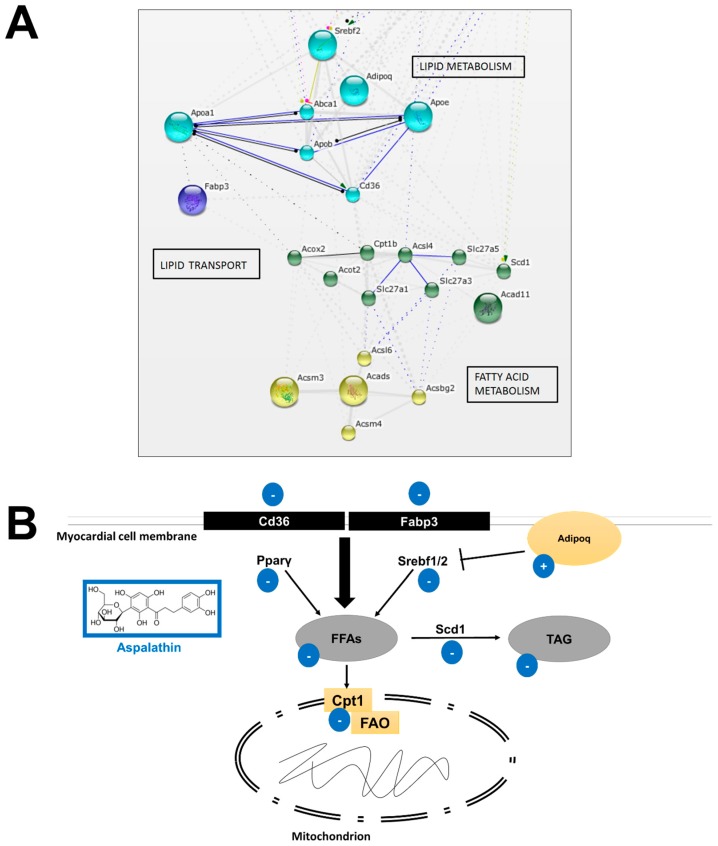
Aspalathin prevented high glucose-induced impaired cardiac substrate metabolism by reducing the uptake and oxidation of free fatty acids. (**A**) Search Tool for the Retrieval of Interacting Genes (STRING) database confirmed a strong interaction between aspalathin treatment and genes associated with lipid transport, lipid and fatty acid metabolism, relevant to dysregulation of intracellular lipid accumulation and fatty acid oxidation; (**B**) Representative diagram of the proposed modulating regulatory mechanisms of aspalathin against increased lipid accumulation and oxidation. *Adipoq*: adiponectin, C1Q and collagen domain containing; *Cd36*: cluster of differentiation 36; *Cpt1*: carnitine palmitoyltransferase 1; *Fabp3*: fatty acid binding protein 3; FAO: fatty acid oxidation; FFAs: free fatty acids; *Pparγ*: peroxisome proliferator activated receptor γ; *Scd1*: stearoyl-Coenzyme A desaturase 1; *Srebf1/2*: sterol regulatory element binding transcription factor 1/2; TAG: triacylglycerides.

To confirm our in vivo findings and to decipher the molecular mechanism used by aspalathin to modulate substrate availability and improve hyperglycemia-associated lipotoxicity, gene expression profiling was performed on H9c2 cardiomyocytes exposed to high glucose. Of the 336 genes assessed, 57 genes (17%) were differentially expressed and of these, 45 and 12 genes (79% and 21%) were hyper-expressed in the high glucose and aspalathin treated groups, respectively (Table 2). Search Tool for the Retrieval of Interacting Genes/Proteins data analysis confirmed that aspalathin treatment largely improved the expression of genes involved in metabolic processes, identifying fatty acid and lipid metabolism as the top two regulatory processes (Figure 1).

#### 2.2.1. In Vitro Effect of Aspalathin on Fatty Acid and Lipid Metabolism

Data analysis identified 26 of the 57 (46%) differentially expressed genes to be involved in fatty acid and lipid metabolism (Table 2). STRING network analysis identified three clusters encoding interactive nodes representing genes associated with fatty acid/lipid transport, lipid metabolism and fatty acid metabolism (confidence score, 0.7) (Figure 1). 

##### Increased β-oxidation

Enhanced free fatty acid (FFA) uptake and lipid storage are causal factors known to precede the development of diabetic heart failure [17,18,19,20]. In this study, we showed that high glucose increased the expression of fatty acid transporters including cluster of differentiation 36 (*CD36*; 3.7-fold), fatty acid-binding proteins 3 (*Fabp3*; 2.7-fold), solute carrier family 25, member 30 (*Slc25a30*; 2.6-fold) and solute carrier family 27, member 1, 3 and 5 (*Slc27a*1; 3 and 5 by 1.0, 6.3-fold and 2.0-fold, respectively) (Figure 1 and Table 2). This increased expression of fatty acid transport was concomitant to raised expression levels of genes associated with β-oxidation including carnitine palmitoyltransferase 1b (*Cpt1b*; 3.3-fold), acyl-CoA thioesterase 2 (*Acot2*; 2.8-fold), acyl-CoA oxidase 2 (*Acox2*; 2.0-fold), as well as lysophospholipase 1 (*Lypla1*; 3.5-fold) (Figure 1 and Table 2). Additionally, we observed that high glucose exposure upregulated mRNA expression of stearoyl-CoA desaturase 1 (*Scd1*; 5.5-fold), an enzyme crucial for the synthesis and storage of fatty acids. However, aspalathin treatment suppressed this effect, which was associated with reduced FFA uptake and oxidation (Figure 1 and Table 2). This result was in agreement with our previous findings [10,21].

##### Increased Supply of Long-Chain Fatty Acids

Chronic hyperglycemia has been associated with an increased supply of circulating FFAs to cardiomyocytes [10,21]. When this enhanced supply of FFAs exceed the rate of β-oxidation, myocardial triglyceride accumulation occurs, leading to lipotoxicity [14,15]. Lipotoxicity subsequently leads to left ventricular dysfunction, a major characteristic feature of DCM. A study done by Drosatos et al. [20] showed that mice with cardiac-specific overexpression of acyl-CoA synthetase (*Acsl*) developed lipotoxicity and diastolic dysfunction, directly implicating long-chain fatty acids (LCFAs) in the development of cardiac fibrosis and subsequent myocardial remodeling [20]. Based on our dataset, STRING analysis identified a network of genes with nodes linking long-chain fatty acyl-CoA synthetase enzymes (Figure 1). These data confirm previous findings [20], where high glucose exposure resulted in increased mRNA expression of acyl-CoA synthetase long-chain family member 4 and 6 (*Acls4*, 5.6-fold and *Acls6*, 2.4-fold) as well as acyl-CoA synthetase medium-chain family member 3 and 4 (*Acsm3*, 14.2-fold and *Acsm*4, 6.1-fold) (Figure 1 and Table 2). Results obtained showed that aspalathin treatment was able to attenuate this effect.

##### Altered Lipid Metabolism and Increased Cholesterol Flux

Scientific evidence has shown that excessive cardiac lipid accumulation contributes to the development of cardiac dysfunction [12,13,22,23]. Lipotoxicity in the myocardium has been associated with the transcriptional factor sterol regulatory element-binding protein 1/2 (*Srebf1/2*) and the transcriptional coactivator peroxisome proliferator-activated receptor-γ (*PPARγ*) [20,22]. *Srebf1/2* and *PPARγ* are important switches that regulate lipid accumulation and lipotoxicity. In a study done by Marfella et al. [15] on biopsies from diabetic patients with left ventricular dysfunction, increased cardiac lipid deposits were concomitant with enhanced mRNA expression of *Srebf1/2*, *PPARγ* as well as genes associated with accelerated β-oxidation. Notably, in this study, elevated levels of circulating FFAs were observed after high glucose exposure. This was parallel to enhanced mRNA expression of *Srebf1/2* (3.4 and 2.0-fold, respectively), as well as *PPARγ* (8.3-fold) (Figure 1 and Table 2). Increased expression of *Srebf1* is further linked to the development of an atherogenic apolipoprotein profile, a characteristic of cardiac hypertrophy [24,25]. Apolipoproteins play an important role in the regulation of lipoprotein metabolism, with the main function being the transport of triglycerides and cholesterol through the lymphatic and circulatory systems. [25]. In particular, *Apoa1* is the major protein component of HDL. HDL promotes efflux of cholesterol, phospholipids, and other lipophilic molecules from cells by an active process mediated by a cell-membrane transporter, ATP-binding cassette transporter (*Abca1*) [23,25]. By contrast, apolipoprotein B (*Apob*) is the main apolipoprotein of chylomicrons and LDL. Elevated LDL levels have been associated with an increased risk of heart failure [23,25]. 

In this study, we observed an increased mRNA expression of *Apob* (7.7-fold), apolipoprotein E (*Apoe*; 4.4-fold) and very low-density lipoprotein (*Vldlr*; 2.0-fold) that was decreased after aspalathin treatment (Figure 1). Like increased HDL cholesterol in *Lepr*^db/db^ mice, *Apoa1* and its transporter *Abca1* were increased by 4.2-fold and 2.0-fold, respectively, after high glucose exposure. Aspalathin treatment reversed this effect. Furthermore, this study showed that high glucose exposure resulted in the reduced expression of *Adipoq* (-6.0-fold), while aspalathin was able to reverse this effect. Cardiac lipotoxicity caused by chronic hyperglycemia may lead to the development of cardiac fibrosis [25,26,27]. We observed that aspalathin treatment was able to reverse lipotoxicity by modulating key regulatory genes, such as *Adipoq*, *PPARγ* and *Sreb1/2* (Figure 1). We propose that aspalathin can prevent lipid accumulation by increasing the expression of *Adipoq.* This increase is associated with a reduced *PPARγ* and *Srebf1/2* expression which may cause lipid accumulation in H9c2 cardiomyocytes. This data was parallel to the observed reduction in systemic total cholesterol, triglycerides and low-density lipoprotein in *Lepr*^db/db^ mice (Figure 1 and Table 2).

#### 2.2.2. In Vitro Effect of Aspalathin on the Development of Insulin Resistance

**Figure 2:**
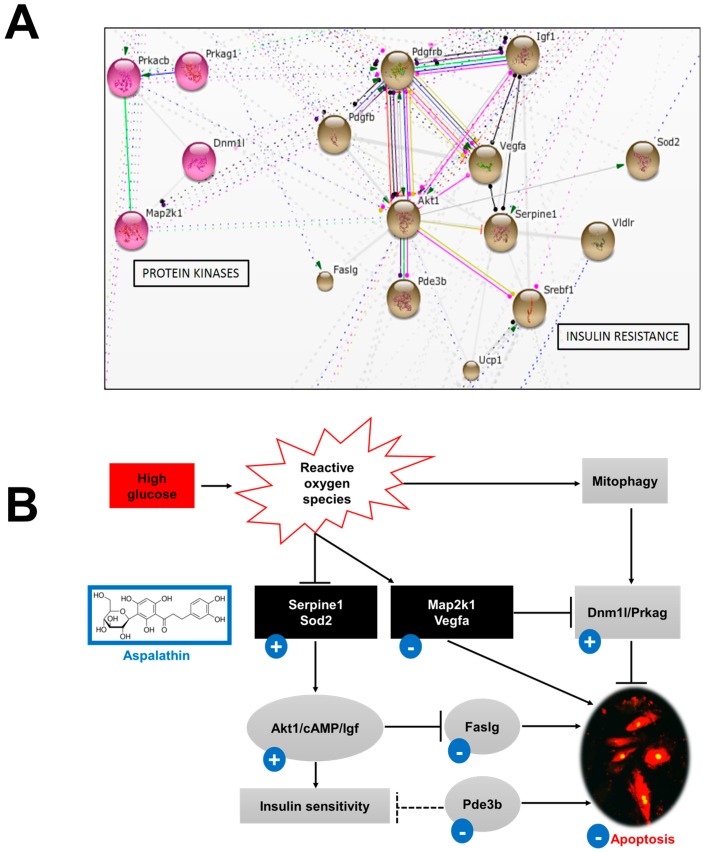
Aspalathin prevented high glucose-induced insulin resistance. (**A**) STRING database analysis confirmed a strong interaction between aspalathin treatment and genes associated with insulin resistance; (**B**) Representative diagram of the proposed protective mechanism of aspalathin against insulin resistance and resultant oxidative stress. *Akt1*: v-akt murine thymoma viral oncogene homolog 1; *cAMP*: cyclic adenosine monophosphate; *Dnm1l*: dynamin 1-like; *Faslg*: fas ligand (TNF superfamily, member 6); *Igf*: insulin-like growth factor 1; *Map2k1*: mitogen-activated protein kinase kinase 1; *Pde3b*: phosphodiesterase 3B; *Prkag*: protein kinase, AMP-activated, γ 1 non-catalytic subunit; *Serpine1*: serpin peptidase inhibitor, clade E (nexin, plasminogen activator inhibitor type 1), member 1; *Sod2*: superoxide dismutase 2; *Ucp1*: uncoupling protein 1; *Vegfa:* vascular endothelial growth factor A.

It is well described that increased LCFAs result in the intramyocardial accumulation of diacylglycerol that activates protein kinase C (θ isoform), leading to the inhibition of insulin receptor substrate 1 and the development of an insulin resistant phenotype [10,11,12]. The latter can reduce cardiac performance as the heart is an insulin-responsive organ. Data analysis revealed that 14 (25%) of the 57 differentially expressed genes were associated with the development of hyperglycemic-induced insulin resistance (Table 2). Network mapping of the differential expressed genes identified two major interconnecting clusters (genes associated with protein kinase activity and development of insulin resistance) with serine/threonine-protein kinase homolog 1 (*Akt1*) and mitogen activated protein kinase (*Mapk*) being the major nodes of connection (confidence score 0.7) (Figure 2).

##### Insulin Signaling and Effect on Myocardium

*Akt1* is a pro-survival protein kinase that plays an important role in the regulation of various cellular functions, including metabolism (glucose and lipids), growth, migration, proliferation and cell survival [28]. STRING network analysis identified *Akt1* as the central node associated with 12 of the 14 differentially expressed genes. *Akt1* constitutes an important node with diverse signaling cascades. In this study, a direct link was observed between phosphodiesterase 3B (*Pde3b*) and *Akt1*. Studies have demonstrated that cAMP-dependent protein kinase signaling is impaired in diabetes and is associated with cardiac dysfunction [29,30]. Cyclic AMP (cAMP) plays a significant role in the thermogenic process, as well as in potentiating glucose-stimulated insulin release [29,30]. In contrast, *Pde3b* is a negative regulator of cAMP and increased expression of this gene inhibits or diminishes the effect of cAMP [23]. *Pde3b* is highly expressed in the myocardium and is known to decrease myocardial smooth muscle contractility [31]. In studies done on isolated rat islets, inhibition of *Pde3b* was reported to improve insulin release [32,33]. In the present study, high glucose exposure resulted in the upregulation of *Pde3b* by 2.1-fold (Figure 1 and Table 2). However, treatment with aspalathin was able to reverse this response. Furthermore, *Akt1* was found to be associated with various antioxidant genes including superoxide dismutase 2 (*Sod2*) and uncoupling protein 1 (*Ucp1*). Aspalathin increased *Sod2* (3.1-fold) expression while the effect of high glucose on *Ucp1* (-1.7-fold) expression was decreased (Figure 2). Interestingly, in a study done by Barreto et al. [34], investigating the mechanism of *Ucp1* action on stress response, they observed that increased *Ucp1* expression induced the upregulation of various antioxidant stress-response genes. We speculate that the observed increased *Ucp1* expression could be a compensatory mechanism used by the cells to ameliorate the adverse effects of increased FFA and hyperglycemia-induced reactive oxygen species (ROS).

Impaired myocardial substrate preference, induced by aberrant FFA levels and insulin resistance, activates a myriad of other maladaptive signaling pathways. Insulin-like growth factor 1 (*Igf1*) activates and phosphorylates *Akt1* to attenuate the development of diabetes-induced myocardial apoptosis, by inhibiting tumor suppressor protein 53 [35,36]. In this study, high glucose had no effect on the expression of *Igf1*, but decreased *Akt1* mRNA expression (-2.2-fold) (Figure 2). Conversely, the tumor necrosis factor ligand superfamily member 6 (*Faslg*) that interacts with *Akt1*, has been shown to induce apoptosis through binding and inhibiting the pro-survival gene CASP8 and FADD-like apoptosis regulator (*Cflar)* [37]. In this study, high glucose treatment increased mRNA expression of *Faslg* (4.8-fold), while aspalathin prevented this response. Thus, our results infer that aspalathin protected cardiomyocytes exposed to chronic hyperglycemia against *Faslg*-induced apoptosis by activating the Igf-PI3K-Akt pro-survival pathway (Figure 2). Additionally, STRING data analysis showed that an interactive network was formed between *Igf1*, *Akt1*, serpin peptidase inhibitor, member 1/2 (*Serpin1/2*) and vascular endothelial growth factor A (*Vegfa*) (Figure 2). 

High glucose treatment resulted in a 2.0-fold increased expression of *Vegfa,* while reducing expression of *Serpinb2* and *Serpine1* by -2.7 and -46.1-fold, respectively (Figure 2 and Table 2). However, we proposed that the observed increased expression of *Serpine1* might lead to cardioprotection; however, this hypothesis requires further investigation as the regulation of *Serpin1* is controversial [38,39,40].

##### Protein Kinases and Mitochondrial Function

Damaged structural components of the heart, caused by insulin resistance, activate signaling pathways such as p38 mitogen-activated protein kinases (*Mapk*) [41]. STRING network analysis identified three distinct nodes within this network, including dynamin 1-like (*Dnm1l*), *Map2k1* and cAMP-dependent protein kinase catalytic subunit (*Prkacb*), that are involved in the process of mitophagy [42]. Roy et al. [43] showed that mice lacking *Dnm1l* demonstrate impaired heart contraction with an associated reduction in mitochondrial fission, concluding that *Dnm1l* is critical for sustaining mitochondrial morphology and heart function. Interestingly, *Dnm1l* levels were increased 2.8-fold after aspalathin treatment. 

Mitophagy induction can also be regulated/activated through the *Prka-MTOR-ULK1* mediated signaling pathway [43]. STRING analysis showed a strong interaction between protein kinase, AMP-activated, γ 1 non-catalytic subunit (*Prkag*) and *Mapk*. In this study we observed that high glucose resulted in the enhanced expression of *Map2k1* (2.3-fold), with an associated decrease in the mitophagy activator *Prkag1* expression (-2.6 fold). However, aspalathin treatment reversed this effect (Figure 2). Thus, we speculate that aspalathin might possibly protect the myocardium from chronic hyperglycemia through enhanced cAMP-mediated mitophagy, thereby alleviating FFA-induced MAPK toxicity and subsequent intracellular lipid accumulation in vitro [44].

#### 2.2.3. In Vitro Effect of Aspalathin on Inflammation

**Figure 3:**
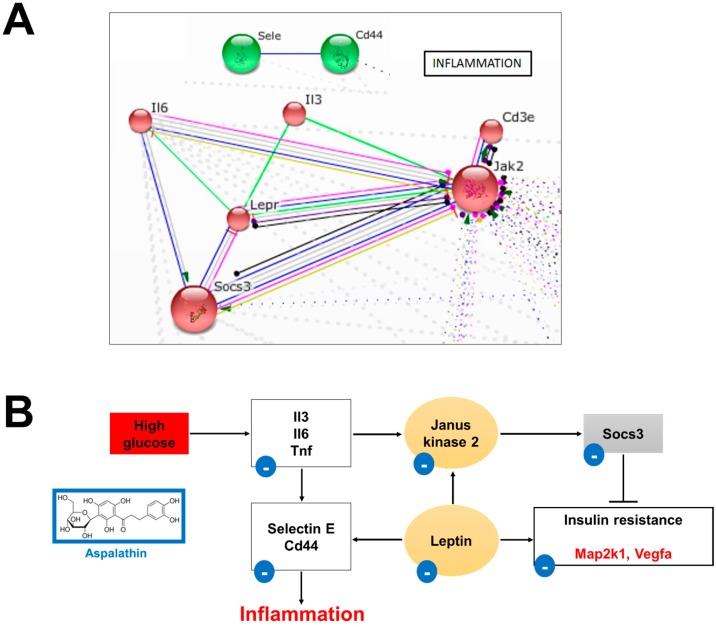
Aspalathin prevented high glucose-induced inflammation. (**A**) STRING database analysis confirmed a strong interaction between aspalathin treatment and genes associated with inflammation; (**B**) Representative diagram of the proposed protective mechanism of aspalathin against high glucose induced inflammation. *Cd44*: cluster of differentiation 44; *Il3*: interleukin 3; *Il6:* interleukin 6; *Map2k1*: mitogen-activated protein kinase kinase 1; *Socs3*: suppressor of cytokine signaling 3; *Tnf*: tumor necrosis factor; *Vegfa*: vascular endothelial growth factor A.

In a diabetic state, a complex interplay between impaired cardiac substrate metabolism, insulin resistance and inflammation underlies the progression of the DCM. Furthermore, evidence is accumulating on the important role of inflammation in the development of cardiac hypertrophy and failure. Aberrant and/or prolonged suppression of cytokine signaling (*Socs*) proteins and Janus kinase (JAK) -induced signaling is detrimental and can give rise to a number of inflammatory pathologies known to affect cardiac function [45,46]. Accordingly, 11 of the 57 (19%) differentially expressed genes were associated with cytokine signaling (Table 2). STRING analysis identified a network with five distinct nodes that include interleukins, Cd3e antigen (*Cd3e*), *Socs3*, and leptin receptor precursor (*Lepr*), with JAK2 (*Jak2*) being the predominant interactive node (Figure 3). 

##### Leptin Signaling

Leptin is an adipokine predominantly expressed in adipose tissue, but it is also expressed in cardiac heart muscle cells [17]. Accumulative evidence suggests that leptin impairs myocardial energy metabolism by favoring a complete reliance on FFAs as an energy source leading to cardiac hypertrophy [17,45]. Leptin binding to the leptin receptor activates the JAK/Signal Transducers and Activators of Transcription (STAT) pathway. Activation of this pathway results in the translocation of *STAT* to the nucleus where it can activate *Socs3*, inhibiting leptin action, as well as insulin signaling [46]. A meta-analysis of our data showed that *Lepr* expression was increased (7.2-fold) after high glucose treatment. Interestingly, this increase was concomitant with an increase in the mRNA expression of both *Jak2* (3.9-fold) and *Socs3* (4.5-fold). This result is of interest, as increased *Lepr* expression is known to enhance *Socs3* with an associated altered β-oxidation [46]. 

##### Cytokine Signaling

In the diabetic heart, increased lipotoxicity and insulin resistance trigger the recruitment of macrophages and leukocytes before the release of pro-inflammatory cytokines such as interleukin 3 and 6 (*Il3* and *Il6*), tumor necrosis factor superfamily (*Tnf*) and cluster of differentiation (*Cd*). *Il6*, *Tnf* and *Cd* cause the endothelial cells of blood vessels to express cellular adhesion molecules, such as Selectin E (*Sele*) and CD4 antigen (*Cd44*), resulting in an acute localized cellular inflammation. Both *CD44* and *Sele* are cell-surface glycoproteins that mediate neutrophil, monocyte, lymphocyte and platelet rolling in the ventricular wall. These glycoproteins also play an important role in atherosclerotic lesion development and calcification of the lesion [47,48]. Studies demonstrated that *Cd44,* as well as *Sele* null mice display atherosclerosis plaque formation [47,48]. Our results showed that high glucose treatment increased the expression of various pro-inflammatory cytokines including, *Il3* (2.3-fold), *Il6* (2.7-fold), *Tnsf13* (4.7-fold) and *Tnfsf13b* (2.1-fold), *Sele* (13.8-fold) and *Cd44* (2.3-fold) (Figure 3 and Table 2). Based on our gene expression data, we propose that during chronic hyperglycemia, pro-inflammatory cytokines (*Il3*, *Il6* and *Tnf-α*) are activated. This increased inflammatory cytokine response releases *Sele* and *Cd44* from the storage granules in activated platelet and endothelial cells and recruits them to mediate the first step of leukocyte extravasation. Additionally, *Il6* and *Tnf* activate the JAK/STAT and Map kinase pathways respectively, resulting in increased hypertrophy and apoptosis. Increased expression of *Socs3* has been linked to inflammation-induced insulin resistance and enhanced JAK/STAT signaling [49]. As observed in this study aspalathin treatment was able to reverse this effect (Figure 3). 

#### 2.2.4. In Vitro Effect of Aspalathin on Apoptosis

Chronic hyperglycemia, insulin resistance and inflammation exacerbates oxidative stress and cardiomyocyte apoptosis [18,21]. In this study, six of the 57 (11%) differentially expressed genes were associated with apoptosis signaling (Table 2). STRING data analysis identified one network with five interactive nodes, centralized around the B-cell lymphoma 2 gene (*Bcl2*).

**Figure 4:**
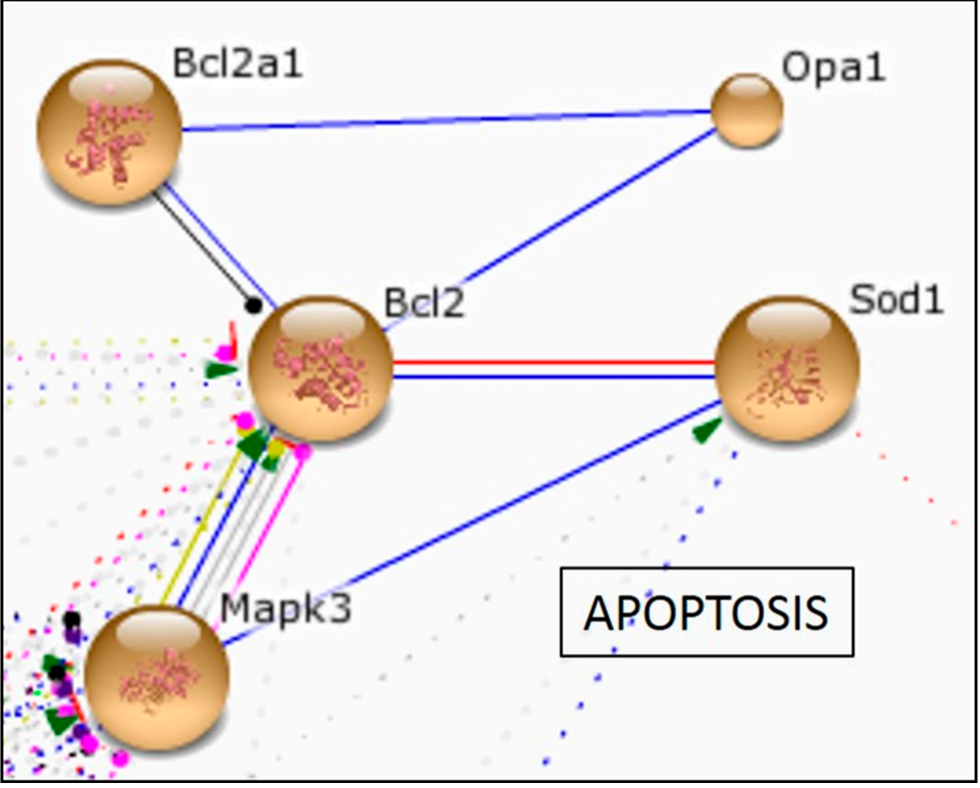
Aspalathin limited high glucose-induced apoptosis. STRING database analysis confirmed a strong interaction between genes associated with aspalathin treatment and regulation of high glucose induced apoptosis.

Genes identified in this network included *Bcl2*, *Bcl2* binding component 3 (*Bbc3*), mitogen activated protein kinase 3 (*Mapk3*) and optic atrophy 1 homolog (*Opa1*) (Figure 4 and Table 2). The *Bcl2* family of proteins are known to be strong modulators of apoptosis and can induce either pro-apoptosis or cell survival depending on the cell’s fate [10,21]. Two pro-survival genes reduced after high glucose exposure were identified (*Bcl2* and *Bcl2a1*). Four pro-apoptotic proteins (*Bbc3*, *Chuk*, *Mapk3* and *Opa1*) were identified (Figure 4 and Table 2). Activation of *Mapk3* together with pro-apoptotic proteins such as *Bbc3* has been associated with myocardial dysfunction [18,50], while upregulation of *Opa1* has directly been linked to the reversal of mitochondrial apoptosis [18,50,51]. Interestingly, aspalathin treatment at a high dose was able to ameliorate this effect by increasing the expression of survival genes. Thus, from this result we proposed that *Bcl2* is the main regulatory network used by aspalathin to protect the myocardium against hyperglycemic-induced cell apoptosis.

### 2.3. Limitations to This Study

Due to tissue limitation, we were unable to test the effect of aspalathin on lipotoxicity markers in heart tissue from *Lepr*^db/db^ mice. However, transcriptional regulation was performed on H9c2 cardiomyocytes, which is an acceptable model to investigate metabolic and signaling alterations associated with cardiac dysfunction.

## 3. Materials and Methods 

### 3.1. Reagents and Kits

Pure aspalathin (ca. 98%, batch SZI-356-54) was synthesized by High Force Research (Durham, UK) according to a previously published protocol [52]. H9c2 rat derived cardiomyoblasts were purchased from the European Collection of Cell Cultures (ECACC No. 8809294; Wiltshire, UK). Radioimmunoassay insulin kit was obtained from Linco Research (St. Charles, MO, USA), halothane from Safeline Pharmaceuticals (Johannesburg, South Africa), Dulbecco’s Modified Eagle’s Medium, penicillin, and streptomycin from Lonza (Verviers, Belgium), and fetal bovine serum sourced from Biochrom (Berlin, Germany). The RNeasy Mini Kit, RT^2^ First Strand Kit, RT^2^ Profiler PCR Arrays and RT^2^ SYBR Green, qPCR Master Mix were obtained from Qiagen (Valencia, CA, USA), while the TRIzol reagent and Turbo DNase Kit were from ThermoFisher Scientific (Waltham, MS, USA). All other consumables and reagents were purchased from Sigma-Aldrich Corp. (St. Louis, MO, USA), unless otherwise specified.

### 3.2. Animal Ethics

Male C57BLKS/J homozygous *Lepr*^db/db^ mice and their heterozygous leptin-receptor-deficient nondiabetic lean littermate controls *Lepr*^db/+^ were obtained from Jackson’s Laboratories (Sacramento, CA, USA) and housed at the Primate Unit and Delft Animal Centre (PUDAC) of the South African Medical Research Council (SAMRC) in a controlled environment with a 12 h light/dark cycle in a temperature range of 23–25 °C (relative humidity: ~50%). The mice received standard laboratory chow pellets (Afresh Vention, Cape Town, South Africa ) *ad libitum* and had free access to drinking water. The study was performed according to principles and guidelines of the South African Medical Research Council’s Guidelines on Ethics for Medical Research: Use of Animals in Research and Training, 2004 (http://ww.mrc.ac.za/ethics/ethicsbook3.pdf) under the institutional ethical approval of the SAMRC (ECRA No. 07/13) as well as Stellenbosch University Ethics Committee (SU-ACUM13-00021).

### 3.3. Aspalathin Treatment of Mice

For this study, animal experiments were only performed to assess the effect of aspalathin on blood lipid profiles. H9c2 cells, as an isolated cell-based system, were used to directly test the effect of aspalathin on the myocardium without the influence of any variables that would be introduced when using an animal model. Following acclimatizing for one week, nine-week old *Lepr*^db/db^ mice together with *Lepr*^db/+^ (*n* = 6 per group) control mice were randomly divided into five groups. Mice were treated daily for six weeks through oral gavage with either a low (13 mg/kg/day) or high (130 mg/kg/day) aspalathin dose and compared to metformin (150 mg/kg/day). Treatment dosages were calculated based on a published study [8]. Treatment groups included: (i) *Lepr*^db/+^ untreated controls (*Lepr*^db/+_UC^); (ii) *Lepr*^db/db^ untreated controls (*Lepr*^db/db_UC^); (iii) *Lepr*^db/db^ treated with metformin (*Lepr*^db/db_MET^); (iv) *Lepr^db/db^* treated with low dose aspalathin (*Lepr*^db/db_ASP_LD^) and (v) *Lepr*^db/db^ treated with high dose aspalathin (*Lepr*^db/db_ASP_HD^). Aspalathin and metformin were dissolved in distilled water before orally administration at the same time (08:00–09:00 a.m.) every day for six weeks, while untreated animals were given water in place of treatment.

### 3.4. Effect of Aspalathin on HOMA-IR 

Mice were fasted for 4 h before plasma glucose levels were measured on a weekly basis by tail pricks using an OneTouch Select handheld glucometer (LifeScan, Milpitas, CA, USA). Fasting plasma insulin was determined using the Radioimmunoassay Kit, as per manufacturer’s instruction. HOMA-IR was calculated using FPG and insulin values, according to a previous described method [53].

### 3.5. Effect of Aspalathin on Lipid Profiles

After the six-week treatment period, 4 h fasted mice were weighed and anesthetized with halothane before blood was collected from the abdominal vena cava for subsequent lipid profile analysis. Blood was centrifuged at 4000× *g* at 4 °C for 15 min before the serum was removed and sent to PathCare Medical Diagnostic Laboratories (Cape Town, South Africa) for total cholesterol, triglycerides, LDL, and high-density lipoprotein (HDL) analyses. 

### 3.6. Cell Culture and Treatment of H9c2 Cardiomyocytes with Aspalathin for Subsequent RT^2^ PCR Profiler Array Analysis

Embryonic ventricular rat heart-derived H9c2 cardiomyoblasts were cultured in supplemented Dulbecco’s Modified Eagle’s Medium (10% fetal bovine serum, 100 U/mL of penicillin, 100 µg/mL of streptomycin) for 48 h under standard tissue culture conditions (37 °C in humidified air and 5% CO_2_). Confluent cells (60%–80%) were seeded at a density of 2 × 10^4^ in 6-well multi-plates. Thereafter, H9c2 cardiomyoblasts were differentiated into adult cardiomyocytes for 7 days using retinoic acid according to a previously described method [54]. On day 8, H9c2 cells were exposed to 33 mM glucose for 48 h and then treated with or without aspalathin (1 µM) for an additional 6 h. Cells exposed to 5.5 mM glucose were used as a normal glucose control [10]. 

### 3.7. RT^2^-PCR Array Analysis of H9c2 Cells Treated with and without Aspalathin

Total RNA from three biological experiments (each with three technical replicates) was extracted using TRIzol reagent, according to a previously described protocol [10]. RNA was purified using the RNeasy Mini Kit (Qiagen, Hilden, Germany), while the Turbo DNase Kit (Ambion, Austin, TX, USA) was used to remove genomic DNA, as per manufacturer’s instructions. RNA integrity was determined using an Agilent 2100 Bioanalyser (Agilent Technologies, Palo Alto, CA, USA). RNA from three independent experiments were pooled and cDNA was synthesized from 2 μg of pooled RNA using the RT^2^ First Strand Kit, according to manufacturer’s instructions. Rat Atherosclerosis (PARN-038ZA-2), Cytokine (PARN-011ZA-2), Fatty Acid Metabolism (PARN-007Z), and Insulin Resistance (PARN-156ZA-2) RT^2^ Profiler PCR Arrays were used for mRNA profiling studies using ABI7500 (ThermoFisher Scientific, Waltham, MA, USA). Analysis of PCR array data was done according to manufacturer’s instructions, using a Microsoft Excel macro available from the manufacturer (http://pcrdataanalysis. sabiosciences.com/pcr/arrayanalysis.php). Each array contained five housekeeping genes (*Actb*, *B2m*, *Gapdh*, *Gusb* and *Hsp90ab1*) against which the sample data were normalized. The transcript level of each candidate gene was quantified according to the ΔΔCt method. Ct values > 35 were not included in the analysis and considered as negative. 

### 3.8. Gene Interaction and Network Analysis

Search Tool for the Retrieval of Interacting Genes/Proteins (STRING; http://string-db.org/) database was used for gene interaction and network analysis to represent information of known and anticipated gene interactions [55,56]. A STRING analysis confidence score of 0.7 was used as a cut off.

### 3.9. Statistical Analysis

Data were expressed as the mean ± SEM. Results for in vitro experiments were expressed as the mean of three independent biological experiments with each experiment containing at least three technical replicates, unless otherwise stated. For in vivo experiments, each treatment group contained six mice. Statistical analysis was performed using GraphPad Prism software version 5.0 (GraphPad Software, Inc., La Jolla, CA, USA). Comparisons between groups were performed using one-way multivariate ANOVA, followed by unpaired Student *t*-test, and a *p*-value of ≤0.05 was designated as statistically significant. 

## 4. Conclusions

Results obtained in this study lead us to propose that aspalathin could protect the myocardium against lipotoxicity in two ways. In the first instance, such treatment significantly improves the blood lipid profile to a far less damaging one therefore attenuating fuel substrate availability to the diabetic heart. It is likely that aspalathin mediates such effects by acting on targets in the liver, although this was not further investigated in the current study. Secondly, our data demonstrate that aspalathin also exerts direct, protective effects within the myocardium to protect the heart against excess fuel substrate availability. Here, our data indicate that aspalathin may protect the myocardium against lipotoxicity and subsequent cell apoptosis by increasing the expression of *Adipoq* and subsequently decreasing the expression of *CD36* and *Cpt1*. Furthermore, aspalathin reduced lipid transport via *Slc27a3/5*, which together with reduced *PPARγ* and *Srebf1* resulted in decreased total cholesterol and subsequent cell apoptosis. Moreover, we proposed that the reversal of lipotoxicity results in a decreased inflammatory response via the *Il6/Jak/Stat* pathway, which together with an observed increase in *Bcl2* prevents myocardium cell apoptosis. Together, these findings proposed a probable mechanism by which aspalathin reverses metabolic abnormalities associated with the failing myocardium. However, this warrants further investigation.

## Figures and Tables

**Table 1:** Effect of aspalathin on blood lipid profiles and HOMA-IR.

Lipid Profile Parameter	*Lepr*^db/+_UC^	*Lepr*^db/db_UC^	*Lepr*^db/db_MET^	*Lepr*^db/db_ASP_LD^	*Lepr*^db/db_ASP_HD^
**Body weight (g)**	27.1±0.33	37.9 ± 0.82 ***	38.0 ± 0.61 ***	37.7 ± 0.74 ***	35.0 ± 0.99 ***^,#^
**Total cholesterol (mmol/L)**	2.4 ± 0.09	3.5 ± 0.18 ***	2.9 ± 0.18 *^,#^	3.2 ± 0.18 **	2.7 ± 0.24 ^#^
**LDL (mmol/L)**	0.1 ± 0.06	0.4 ± 0.07 *	0.2 ± 0.04 ^#^	0.2 ± 0.05	0.1 ± 0.04 ^##^
**HDL (mmol/L)**	1.7 ± 0.06	2.5 ± 0.10 ***	2.2 ± 0.13 **	2.3 ± 0.13 **	2.3 ± 0.14 **
**Triglycerides (mmol/L)**	0.9 ± 0.04	3.2 ± 0.38 ***	2.3 ± 0.46 *	2.6 ± 0.19 ***	2.3 ± 0.28 **
**Insulin (ng/mL)**	0.4 ± 0.09	1.9 ± 1.02	1.1 ± 0.22	1.5 ± 0.34	0.7 ± 0.10
**FPG (mmol/L)**	5.6 ± 0.29	21.2 ± 1.34 ***	18.1 ± 1.68 ***	22.2 ± 1.50 ***	22.0 ± 1.41 ***
**HOMA-IR**	0.1 ± 0.01	0.6 ± 0.28	0.3 ± 0.05	0.4 ± 0.07	0.2 ± 0.03

Results are the mean ± SEM, with each treatment group containing six mice. * *p* < 0.05, ** *p* < 0.001, *** *p* < 0.0001 versus heterozygous leptin-receptor-deficient (*Lepr*) nondiabetic lean littermate untreated controls (*Lepr*^db/+_UC^); ^#^ *p* < 0.05, ^##^ *p* < 0.001 versus homozygous leptin-receptor-deficient diabetic mice untreated controls (*Lepr*^db/db_UC^). *Lepr*^db/+_ASP_LD^: diabetic mice treated with low dose aspalathin (13 mg/kg), *Lepr*^db/+_ASP_HD^: diabetic mice treated with high dose aspalathin (130 mg/kg), *Lepr*^db/+_MET^: diabetic mouse treated with metformin (150 mg/kg), FPG: fasting plasma glucose, HDL: high density lipoprotein, HOMA-IR: homeostasis model assessment: insulin resistance, LDL: low density lipoprotein.

**Table 2:** Effect of aspalathin on the transcriptional profile of genes involved in metabolic processes.

Gene Name	Gene Symbol	Gene Fold Regulation	
		High Glucose (33 mM)	Aspalathin (1 µM)
**Lipid metabolism**			
ATP-binding cassette, subfamily A (ABC1), member 1	*Abca1*	2.0776	−1.1921
Acyl-Coenzyme A dehydrogenase, C-2 to C-3 short chain	*Acads*	−1.6611	−2.425
Acyl-CoA thioesterase 2	*Acot2*	2.8239	−1.2178
Acyl-Coenzyme A oxidase 2, branched chain	*Acox2*	2.0959	1.8895
Acyl-CoA synthetase bubblegum family member 2	*Acsbg2*	4.3205	−1.5973
Acyl-CoA synthetase long-chain family member 4	*Acsl4*	5.6547	1.7361
Acyl-CoA synthetase long-chain family member 6	*Acsl6*	2.465	−1.5973
Acyl-CoA synthetase medium-chain family member 3	*Acsm3*	14.2353	5.697
Acyl-CoA synthetase medium-chain family member 4	*Acsm4*	6.1301	3.1806
Adiponectin, C1Q and collagen domain containing	*Adipoq*	−6.0324	3.2588
Apolipoprotein A-I	*Apoa1*	4.2006	2.9178
Apolipoprotein B	*Apob*	7.7651	−3.0228
Apolipoprotein E	*Apoe*	4.4207	−1.0317
CD36 antigen	*Cd36*	3.7512	1.7387
Carnitine palmitoyltransferase 1b, muscle	*Cpt1b*	3.3366	2.0449
Fatty acid binding protein 3, muscle and heart	*Fabp3*	2.7003	−1.5135
Lysophospholipase 1	*Lypla1*	3.5198	1.6047
Peroxisome proliferator activated receptor gamma	*Ppar*γ	8.3847	−1.6135
Stearoyl-Coenzyme A desaturase 1	*Scd1*	5.5982	1.0879
Solute carrier family 25, member 30	*Slc25a30*	2.6123	1.6634
Solute carrier family 27 (fatty acid transporter), member 1	*Slc27a1*	1.0514	−2.2919
Solute carrier family 27 (fatty acid transporter), member 3	*Slc27a3*	6.323	2.2207
Solute carrier family 27 (fatty acid transporter), member 5	*Slc27a5*	2.0184	−1.5973
Sterol regulatory element binding transcription factor 1	*Srebf1*	3.4573	−4.544
Sterol regulatory element binding factor 2	*Srebf2*	2.0208	−26.826
Very low density lipoprotein receptor	*Vldlr*	2.0148	1.3077
**Insulin resistance**			
V-akt murine thymoma viral oncogene homolog 1	*Akt1*	−2.2173	1.2477
Dynamin 1-like	*Dnm1l*	1.4367	2.8522
Fas ligand (TNF superfamily, member 6)	*Faslg*	4.8622	−1.4856
Insulin-like growth factor 1	*Igf1*	1.3447	2.4477
Mitogen-activated protein kinase kinase 1	*Map2k1*	2.3446	−2.1822
Phosphodiesterase 3B, cGMP-inhibited	*Pde3b*	2.1481	1.5951
Protein kinase, cAMP dependent, catalytic, β	*Prkacb*	1.687	2.4466
Protein kinase, AMP-activated, γ 1 non-catalytic subunit	*Prkag1*	−2.6381	1.3516
Serpin peptidase inhibitor, clade B (ovalbumin), member 2	*Serpinb2*	−2.7789	1.8746
Serpin peptidase inhibitor, clade E (nexin, plasminogen activator inhibitor type 1), member 1	*Serpine1*	46.1814	−1.4005
Superoxide dismutase 1, soluble	*Sod1*	2.8805	1.0032
Superoxide dismutase 2, mitochondrial	*Sod2*	−1.5522	3.1195
Uncoupling protein 1 (mitochondrial, proton carrier)	*Ucp1*	58.6622	−1.7821
Vascular endothelial growth factor A	*Vegfa*	2.0015	1.2082
**Inflammation**			
CD3 antigen, epsilon polypeptide	*Cd3e*	5.4019	1.3557
CD44 molecule	*Cd44*	2.3883	1.322
Interleukin 3	*Il3*	2.3815	−1.1606
Interleukin 6	*Il6*	2.7362	1.9106
Janus kinase 2	*Jak2*	3.9723	1.721
Leptin receptor	*Lepr*	7.2781	−1.6135
Selectin E	*Sele*	13.8787	−1.5233
Suppressor of cytokine signalling 3	*Socs3*	4.5848	−2.4959
Tumor necrosis factor receptor superfamily, member 1b	*Tnfrsf1b*	1.0099	−3.114
Tumor necrosis factor (ligand) superfamily, member 13	*Tnfsf13*	4.7142	1.2849
Tumor necrosis factor (ligand) superfamily, member 13b	*Tnfsf13b*	2.0522	1.8583
**Apoptosis**			
Bcl-2 binding component 3	*Bbc3*	−1.3703	−3.1586
B-cell CLL/lymphoma 2	*Bcl2*	−2.6947	1.8312
B-cell leukemia/lymphoma 2 related protein A1d	*Bcl2a1*	−7.13	1.8085
Conserved helix-loop-helix ubiquitous kinase	*Chuk*	4.2959	2.1502
Mitogen-activated protein kinase 3	*Mapk3*	1.2583	−3.0871
Optic atrophy 1 homolog (human)	*Opa1*	1.3949	2.0255
